# Monitoring of Heavy Metals and Pesticide Residues of Herbal Decoctions in Traditional Korean Medicine Clinics in Korea

**DOI:** 10.3390/ijerph19148523

**Published:** 2022-07-12

**Authors:** Hye In Jeong, Ji-Eun Han, Byung-Cheul Shin, Soo Bin Jang, Jae-Hee Won, Kyeong Han Kim, Soo-Hyun Sung

**Affiliations:** 1 Department of Preventive Medicine, College of Korean Medicine, Kyung Hee University, Seoul 02447, Korea; frogcream@gmail.com; 2 Department of Policy Development, National Institute of Korean Medicine Development, Seoul 04554, Korea; jieun2342@nikom.or.kr; 3 Division of Clinical Medicine, School of Korean Medicine, Pusan National University, Yangsan 50612, Korea; drshinbc@pusan.ac.kr; 4 Department of Preventive Medicine, College of Korean Medicine, Daegu Haany University, Gyeongsan 38609, Korea; suebin@nate.com; 5 Department of Public Infrastructure Operation, National Institute of Korean Medicine Development, Seoul 04554, Korea; won10042@nikom.or.kr; 6 Department of Preventive Medicine, College of Korean Medicine, Woosuk University, Jeonju 54986, Korea

**Keywords:** herbal decoction, quality control, safety management standard, heavy metals, pesticides

## Abstract

In this study, we investigated whether the levels of heavy metal and pesticide residues in herbal decoctions in Korea in 2019 were within normal limits. In total, 30 decoctions composed of multi-ingredient traditional herbs were sampled from traditional Korean medicine (TKM) clinics, TKM hospitals, and external herbal dispensaries in 2019. The decoctions were analyzed for heavy metal content such as lead, arsenic, and cadmium using inductively coupled plasma optical emission spectrometry. For mercury, an automatic mercury analyzer based on the gold amalgamation process was used. For pesticide residues, gas chromatography with electron capture detection and gas chromatography with mass selective detection were used for the analyses. Based on the testing, heavy metals were identified in most of the decoctions (Cd: 0.000–0.003 ppm, Pb: 0.003–0.023 ppm, As: 0.000−0.016 ppm, Hg: 0.000–0.002 ppm). Pesticide residues (e.g., total DDT, total BHC, aldrin, endrin, dieldrin) were not detected at all. All of these were no more than the limit values in preceding studies. Therefore, this study confirms that the contents of heavy metals and pesticides in herbal decoctions are within safe levels based on a previous study and provides evidence for establishing safety management standards for herbal decoctions in Korea.

## 1. Introduction

Currently, the demand for traditional and complementary medicine (T&CM) is increasing [1]. Herbal medicine, the popular one of T&CM, has been widely used globally. More than one-third of adults reported using at least 1 herbal medicine in the USA [2]. The global herbal medicine market size was estimated to be US $83 billion in 2019 and is expected to reach US $550 billion by 2030 [3]. In fact, 80% of the world’s total population has been reported to depend on herbal medicines for primary health care [4].

However, raw plants, which are the main ingredients of herbal medicines, are easily contaminated by various materials since they assimilate the elements in soil quickly through their roots [5]. Especially, some heavy metals, such as lead, mercury, and cadmium, can cause poisoning if taken even in small amounts for a long time [6]. In biological systems, heavy metals have been reported to affect cellular organelles and components such as the cell membrane, mitochondria, lysosomes, endoplasmic reticulum, nuclei, and some enzymes involved in metabolism, detoxification, and damage repair [7]. Metal ions play an important role in the interactions between cell components. Therefore, intakes of heavy metals may lead cell cycles to modulation, carcinogenesis, and apoptosis [7,8,9]. Various human-health-related concerns are associated with pesticides, ranging from short-term impacts such as headaches and nausea to chronic impacts such as various cancers, birth defects, infertility, and endocrine disruption [10,11].

In the United States, these issues caused the United States Environmental Protection Agency to develop detailed step instructions in order to assess potential health risks [12]. As for Europe, the Committee on Herbal Medicinal Products introduced a program called the Traditional Use Registration [13].

In Korea, herbal medicine manufactured by good manufacturing practice (GMP) company used in the Traditional Korea Medicine (TKM) institution, and the amounts of hazardous substances (e.g., heavy metals, pesticides, aflatoxins, sulfur dioxide, and benzopyrene) in medicinal herbs are restricted by the Regulations on Limits and Test Methods for Residues and Contaminants in Herbal Medicines. TKM institutions in Korea must use medicinal herbs manufactured by the herb-GMP (hGMP) facilities that are licensed by the Ministry of Food and Drug Safety (MFDS) for safety [14]. However, the decoction type of herbal medicines (DHM), which is combined with two or more medicinal herbs, lacks a safety management standard in TKM clinics where those DHM are dispensed [15].

Seo [16] reported that almost no heavy metals and pesticide residues were detected when they compared five medicines for the common cold before and after decocting for heavy metals and pesticide residue contents. Yu et al. [15] investigated heavy metals, pesticides, and sulfur dioxide in 155 traditional herbal decoctions. Their average concentrations (77.0 ± 79.7 µg/kg for As, 20.4 ± 23.7 µg/kg for Cd, and 68.8 ± 76.5 µg/kg for Pb) were approximately 20% of the maximum allowable limits for vegetable or ginseng beverages described in the Korean Food Standard Codex. None of the 33 pesticides were detected in 155 decoction samples.

Since 2020, the South Korean government has been implementing pilot programs to provide health care coverage to herbal decoctions. Despite decoctions being proved safe in history, there is no system to verify the safety of herbal decoctions, and there is a national demand for verification. Accordingly, the government is taking a leading role in implementing a project to establish the safety management criteria for herbal decoctions. The authors monitored the quality control of herbal decoctions as part of this project. As herbal decoctions are prepared in the three facilities (TKM clinics, hospitals, and external herbal dispensaries, which is a type of pharmacy that provides various types of herbal medicines to other TKM institutions) in Korea, the researchers collected the 10 most commonly used herbal decoctions in TKM institutions from TKM clinics, TKM hospitals, and external herbal dispensaries (30 decoctions in total). In this study, we aimed to monitor the decoctions in Korea for heavy metal and pesticide residue contents and to prepare the evidence for establishing management standards at the government level.

## 2. Materials and Methods

### 2.1. Sample Collection

Based on the TKM Consumption Statistics Survey 2017, which is the official government statistics data, the researchers tested ten different decoctions, including Galgeuntang, which are prescribed commonly in TKM institutions to examine them for any heavy metal or pesticide residue contents [17]. For each prescription over the period of October to December 2019, the researchers secured 30 herbal decoction pouches from TKM clinics, TKM hospitals, and external herbal dispensaries in pouches and stored then in a refrigerator to use them in tests. The 10 types of herbal decoction formulae and compositions are shown in Table S1 in Supplementary Materials. The 10 types of herbal decoctions are prepared in TKM clinics, TKM hospitals, or external herbal dispensaries without registration with the MFDS. The individual herbs that compose the 10 decoctions are registered in the MFDS.

### 2.2. Standards and Reagents

For the heavy metal standard, the 1000 mg/kg (SCP Science, Baie-D'Urfé, QC, Canada) product was used. The standards for lead, arsenic, and cadmium were diluted using 0.5 M nitric acid (Merck, Germany) before use. The standard for mercury was diluted using 0.001% l-cysteine (98%, Nacalai Tesque Inc., Kyoto, Japan). HG-MHT and HG-BHT (NIC., Japan) were used as the additives for the analyses of mercury.

The standards used for testing pesticide residues were α-benzene hexachloride (BHC), β-BHC, γ-BHC, δ-BHC, aldrin, dieldrin, endrin, P.P-dichlorodiphenyldichloroethane (DDD), P.P-dichlorodiphenyldichloroethylene (DDE), O.P-dichlorodiphenyltrichloroethane (DDT), and for P.P-DDT, Dr. Ehrenstorfer (LGC Standards GmbH, Wesel, Germany) products were used. The solvents used in extraction and refining were acetone and methylene chloride, and n-hexane from Merck was used for pesticide residue analysis, while the Florisil Cartridges (1000 mg, 6 mL) for refining were from Waters (USA).

### 2.3. Experimenting Methods

#### 2.3.1. Pre-Treatment of Samples

For the testing of heavy metals, the testing solution was prepared in accordance with Korea Pharmacopoeia (KP) General Testing Process 30. Botanical Medicine Testing Method Item B. Heavy Metals Testing Method. As for the pesticide residues, KP General Testing Process 30. Botanical Medicine Testing Method Item C. Pesticide Residues Testing Method was used as the basis for preparing testing solutions [18].

For some heavy metals, namely lead, arsenic, and cadmium, 10.0 mL of the sample was mixed with 20 mL of nitric acid, before standing for 30 min to remove the emitting gas. Subsequently, a wet pyrolysis device was used to conduct pyrolysis before it is cooled down to room temperature. The separated liquid was corrected to 50 mL using distilled water as the test solution. Separately, nitric acid 20 mL was prepared in the same way and was used as the blank test solution. For mercury, which is a heavy metal, 50 µL was measured exactly and added to the vessel for mercury analysis in the order of M reagent → sample (50.0 µL → M reagent → B reagent → M reagent) before conducting the analysis.

As for the analysis of pesticide residues, 10.0 mL of sample was added to 15 mL of distilled water and 90 mL of acetone before homogenization and vacuum filtration. This filtered solution was transferred to a 500 mL separating funnel followed by the addition of saturated sodium chloride solution, 50 mL distilled water, 100 mL dichloromethane, and 70 mL and shook rapidly to mix. Then, the solution was left to stand for stratification. The dichloromethane layer was gathered, and 70 mL dichloromethane was added to the water layer and extracted for mixing with the dichloromethane layer. Next, this mixture underwent dewatering filtration with sodium sulfate anhydrous. The filtered solution was vacuum-evaporated at <40 °C and melted in 4 mL of hexane. For refining, the solution was added to an activated Florisil cartridge and extracted using hexane–dichloromethane–acetone mixture (50–48.5–1.5 mL). The extracted solution was added with 5 mL and melted in a 2-mL mixture of hexane and acetone (8:2) to conduct the analysis.

#### 2.3.2. Analysis Instrument and Instrument Conditions

For the analysis of lead, arsenic, and cadmium, an inductively coupled plasma optical emission spectrometer (ICP-OES, iCAP6500 Thermo, Waltham, MA, USA) was used. The instrument analysis conditions are presented in Table 1. Of the heavy metals, mercury was analyzed using an automatic mercury analyzer (Model MA-2, Nippon Instrument Co., Kyoto, Japan), which is based on gold amalgamation process. The analysis conditions of the instrument are presented in Table 2. The analysis of pesticide residues was conducted using gas chromatography with electron capture detection (GC/ECD, Agilent 7890, Santa Clara, CA, USA) and gas chromatography with mass selective detection (GC/MSD, Agilent 5975, Santa Clara, CA, USA), the analysis conditions of which are presented in Table 3.

### 2.4. Validation of the Test Method

The recovery rate was measured as follows. First, a sample from which no analysis material was detected was selected. Then, a standard solution of appropriate concentration was added, and this process was repeated three times. With this, the difference between the concentration of the samples containing standard and the concentration of the control was calculated. The limit of detection (LOD) and the limit of quantitation (LOQ) were calculated using the following formula, on top of the method in which the standard deviation of the reaction and the gradients of the measurement graph were used as the basis and the measurement graph was based on the average of three repetitions of measurement of the standard solution in a stepwise manner. Recovery rate, LOD, and LOQ of analysis equipment are listed in Table 4.

LOD = 3.3 × σ/S LOQ = 10 × σ/S

* σ: the mean standard deviation

* S: the individual slop

### 2.5. Test Criteria

The test items and safety management criteria for heavy metals and pesticide residues were developed in accordance with the findings in literature reviews and consensus between experts. More details on the development process are presented in a previous study [19]. As for heavy metals, the contents of lead, arsenic, cadmium, and mercury were included in the criteria. Specifically, lead should be ≤5 ppm, arsenic ≤ 3 ppm, cadmium ≤ 0.3 ppm, and mercury ≤ 0.2 ppm. As for pesticide residues, the contents of five organic chlorines were used as the criteria, namely total DDT ≤ 0.1 ppm, total BHC ≤ 0.02 ppm, and aldrin, endrin, and dieldrin should all be ≤0.01 ppm [19].

## 3. Results

### 3.1. Heavy Metals

In the analysis of 30 samples for their lead, arsenic, cadmium, and mercury contents, heavy metal contents were below the criteria in all the samples (Table 5). The detection amount of 30 decoctions was 0.046 ± 0.0007 ppm for cadmium, 0.3 ± 0.004 ppm for lead, 0.207 ± 0.004 ppm for arsenic, and 0.026 ± 0.0005 ppm for mercury.

### 3.2. Pesticide Residues

Regarding total DDT, total BHC, aldrin, endrin, and dieldrin contents, pesticide residues were less than the detection limit in all of the samples.

## 4. Discussion

In the researchers’ previous study [19], a set of safety management criteria was identified through a review of existing literature (e.g., WHO guidelines for the regulation of herbal medicines, pharmacopeia of the people’s Republic of China, Japanese pharmacopeia, Japanese standards for nonpharmacopeial crude drugs, European pharmacopeia, A quality control guideline for herbal or botanical medicine extracts of Korea, and the US botanical drugs guidance) and consensus between experts on heavy metals, pesticide residues, sulfur dioxide, benzopyrene, mycotoxin, and micro-organism limits.

In this study analyzing the heavy metal content in 30 decoctions, most of them contained heavy metals (Cd: 0.000–0.003 ppm, Pb: 0.003–0.023 ppm, As: 0.000−0.016 ppm, Hg: 0.000–0.002 ppm). As the detection limits identified in the previous study for TKM decoctions safety management criteria were, respectively, lead 5 ppm, arsenic 3 ppm, cadmium 0.3 ppm, and mercury 0.2 ppm, none of the 30 samples exceeded the criteria in any heavy metal items. Here, the detected levels of As, Cd, and Pb were lower than those in the previous study [15] (As 0.0–0.5824 ppm (mg/kg), Cd: 0.0–0.219 ppm (mg/kg), Pb: 0.0–0.6317 ppm (mg/kg)). Hg was not detected in the preceding study. However, in this study, Hg was detected in some of the samples [15]. The Korean MFDS introduced the hGMP in 2012 and made it mandatory in 2015. Thus, medicinal herbs must be manufactured by the hGMP facilities that are licensed by the Korean MFDS [13]. The comparison of the heavy metal contents in the herbal medicines before decocting and the decoctions of these herbal medicines indicated that Pb decreased by 94.9%, Cd by 97.3%, As by 92.5%, and Hg by 98.1% [20]. Hence, almost no heavy metals were detected in the decoction formulation, in addition to the management of heavy metals in each herbal medicine and the low herbal medicine heavy metals transfer rate of ≤10%.

Since the cases of heavy metal poisoning by herbal medicines are reported continuously, it can be considered an important issue [21]. On analyzing in terms of heavy metals, lead, arsenic, cadmium, and mercury were selected because they are frequently detected in the processing period of herbal medicines [21,22]. Researchers in the countries where herbal medicines are in use are continuously conducting studies that monitor heavy metals and pesticide residue contents in herbal medicines [23,24]. In a review study on heavy-metal-related adverse events conducted in South Korea, many cases of lead poisoning involving stomach pain, along with some cases of arsenic and cadmium poisoning, have been reported [22]. The chemical composition and the contents of substances in herbal medicines vary widely depending on their origin, environment of growth, place of production (wild or cultivated), and time of harvest. Therefore, the quality of the extracts or the formulations that are made from herbal medicines (botanical medicines) primarily depend on the quality of the herbal medicines (botanical medicine), the substances and their contents in the extract, the production process, which is directly related to yield rate, and the management of the standard to maintain consistency in quality. Therefore, implementing an integrated quality management is necessary to minimize the variations in terms of substances and the contents of the raw herbal materials and their extracts [25].

None of the 30 decoctions contained any of the pesticide residues test items (e.g., total DDT, total BHC, aldrin, endrin, and dieldrin). In Seo’ s study [16], no pesticide residues were discovered in the herbal medicines before decocting and the decoction. According to a Chinese study, washing, carbonizing, and boiling reduced pesticide residues significantly. The effects of pesticide were reduced by 41.2–60% by washing and 27.1–71.1% by carbonizing, while boiling practically removed or literally removed any pesticide residues [26]. Although the risk of detecting pesticide residues in herbal decoctions can be considered low, herbal products of various countries require more than 380 pesticides. [27,28]. Korea also requires screening of 464 pesticides for agricultural products, and the 30 decoctions analyzed by the authors are medical productions used by TKM doctors and follow KP standards. Therefore, the authors performed the analysis according to the test method of five organic chlorine-based pesticides (e.g., DDT, BHD, aldrin, dieldrin, and endrin) determined by KP. In the future, monitoring of herbal decoctions based on agricultural and KP standards, it is necessary to verify whether there are pesticides to be further monitored through comparative analysis studies. Although today’s proper use of pesticides is safe and can improve the yield and quality of `the agricultural products, concerns on potential risks associated with pesticide use abound [18]. Moreover, the water solubility and partition coefficient values of the pesticides may have played a basic role in the dissipation of the residues during the decocting process [29]. Pesticide residues can be as harmful as heavy metals to human health. The test items were DDT, BHC, aldrin, endrin, and dieldrin. These are all persistent organic pollutants, and at the same time, organochlorine pesticide which can build up in the human body [30]. They disrupt the endocrine system to increase the risk of cancers. They may also likely cause diabetes, obesity, cardiovascular diseases, and other metabolic diseases [31].

South Korea is a member state of the Pharmaceutical Inspection Cooperation Scheme (PIC/S), and is required to follow the GMP, good laboratory practice (GLP), and good clinical practice (GCP) guidelines [32,33]. These apply to the safety, efficacy, and quality management of the herbal medicine and herbal material manufactured by pharmaceutical companies at all times. In PIC/S guide to good manufacturing practice for medicinal products annexes, manufacture of herbal medicinal products was recorded. This annex applied to all herbal starting materials: medicinal plants, herbal substances, or herbal preparations. We compared the PIC/S guide with hGMP and found several differences as follows. First, in PIC/S, the equipment must be compatible with the extraction solvent to prevent any release or undesirable absorption of substance. Although hGMP had no exact clause about using proper solvent, a similar one, regulation on machine used in manufacturing process, was documented. Second, there was a clause about herbal preparation in PIC/S, but there was not one in hGMP. Because it was limited to herbs, such as *Paeonia lactiflora*, and there was a regulation on herbal preparation separately, it was of little importance. PIC/S emphasized that regulation should be determined in accordance with the relevant current national or international guidance on quality. As we mentioned before, South Korea is required to follow several practice guidelines, so the regulation in South Korea should follow the international guidance quickly.

In the study of Yu et al. [18], 155 decoctions were randomly collected from TKM clinics, herbal pharmacies, and herbal medicine shops. Herbal pharmacies and herbal medicine shops are places that sell herbal products regardless of the prescription of a TKM doctor. Therefore, it was insufficient to be used as an appropriate basis for preparing safety management standards for herbal medicines as pharmaceuticals. In our study, 10 types of herbal decoctions prescribed by a TKM doctors were collected and monitored from each of TKM clinic, TKM hospital, and external herbal dispensary. The three types of facilities that collect decoctions represent the route of preparing herbal medicine as a medical product. In addition, this study was initiated to establish safety management standards for medical herbal decoctions according to the official project of the Korean government. Therefore, the results of this study are not simply monitoring results but will contribute as evidence to establish safety management standards for herbal decoctions.

Our study had several limitations. First, the number of the samples included in the analyses insufficient to make this study available as the supporting evidence to introduce the standard set in the preceding study as a legal requirement. Thus, gathering and analyzing more samples in the future are necessary in order to verify the safety management criteria. Second, only the heavy metals and pesticide residues were analyzed, and sulfur dioxide, benzopyrene, aflatoxin, and micro-organism limits should also be analyzed. According to KP [19], *Rehmanniae radix preparata* (熟地黃) and *Rehmanniae radix* (地黃) had the standards for testing benzopyrene. According to Yoon et al. [34], 46.8% of the domestic or imported herbal medicines (41 categories, 235 samples) contained benzopyrene. Since herbal medicines are not verified for benzopyrene content, verifying the final products (decoction) for their benzopyrene contents is absolutely necessary, for which further study is mandated. Third, it was difficult to guarantee representativeness of the sample because the number of samples was small and regional distribution was not considered during the sampling process. In the future, sampling that represent herbal decoctions samples of Korea should be conducted in consideration of factors such as regional distribution and number of samples. Finally, the sensitivity, accuracy, and precision of the analysis of heavy metals and pesticide residues in different decoction were not performed in this study. The heavy metal and pesticide residue test method was based on the Korean Pharmacopeia, and NIKOM, which conducted the monitoring and analysis, was approved by Korea MFDS as an institution that can test herbal materials and herbal medicines. Although the accuracy and reliability of the test results are at a certain level, sensitivity, accuracy, and precision of the analysis should be performed in future research.

## 5. Conclusions

Our study presents the status of herbal decoctions regarding the contamination with heavy metals and pesticides, which are within the safe range in Korea. These data provide the supporting evidence to set the safety management criteria for decoction. The researchers hope that more samples would be gathered and analyzed for their heavy metal and pesticide residue contents in order to establish the safety management criteria and successfully introduced a program on this.

## Figures and Tables

**Table 1:** Analysis conditions of ICP-OES.

Instrument	Analysis Condition
Power	1.15 kW
Plas. Flow	12.0 L/min
Aux Flow (L/min)	0.50 L/min
Neb.Flow (L/min)	0.50 L/min
Replicate Time (s)	5.00 s
Stable Time (s)	15 s
Sample uptake (s)	50 s
Rinse Time (s)	10 s
Pump Rate (rpm)	50 rpm
Fast pump	on
Replicates	3

ICP-OES: inductively coupled plasma optical emission spectroscopy.

**Table 2:** Analysis conditions of automatic mercury analyzer.

Instrument	Analysis Condition
Method	Gold amalgam collection
Wavelength(nm)	253.7
Mode selector	Standard: 1, Sample: 2
Combustion tube	Filling catalysts
Flow meter	0.5 L/min
Carrier gas	Purified dry air
Heating mode	Two available modes
Heating temperature	850 °C
Measuring range	0∼1000 ng
Gas washing bottle	Buffer solution of pH 6.86

**Table 3:** Analysis conditions of GC/ECD and GC/MSD.

Instrument					Analysis Condition		
Detector	GC/ECD				GC/MSD		
Column	DB-5 (50 m × 0.25 mm × 0.25 µm)				DB-5MS (30 m × 0.25 mm × 0.25 µm)		
Oven temperature	Rate (°C/min)	Temp. (°C)	Hold (min)	Time (min)	Rate (°C/min)	Temp. (°C)	Hold (min)
	Initial	80	2	2	Initial	80	2
	20	200	5	13	10	180	3
	1	230	3	46	2	220	2
	20	290	20	69	10	280	20
Inject temperature	250 °C				250 °C		
Detector temperature	290 °C				290 °C (Interface)		
Injection volume	1 µL (10:1)				0.8 µL (splitess)		
Flow rate	0.8 mL/min				1.0 mL/min		

GC/ECD: gas chromatograph with electron capture detection, GC/MSD: gas chromatography with mass selective detection.

**Table 4:** Recovery rate, detection limit, and quantitation limit of analysis equipment.

		Recovery Rate (%)	Limit of Detection (ppm)	Limit of Quantitation (ppm)
Heavy metals	Pb	92.9	0.0015	0.0045
	As	95.9	0.0021	0.0064
	Cd	99.5	0.0001	0.0003
	Hg	100.4	0.0006	0.0018
Pesticide residues	α-BHC	98.1	0.01	0.03
	β-BHC	94.3	0.01	0.03
	γ-BHC	95.7	0.01	0.03
	δ-BHC	94.4	0.01	0.03
	Aldrin	74.1	0.005	0.01
	Dieldrin	88.5	0.005	0.01
	Endrin	85.9	0.005	0.01
	p.p-DDE	82.2	0.01	0.03
	p.p-DDD	81.9	0.01	0.03
	o.p-DDT	85.3	0.01	0.03
	p.p-DDT	92.9	0.01	0.03

**Table 5:** Heavy metal detection result of herbal decoctions.

Herbal Decoctions		Cd (ppm)	Pb (ppm)	As (ppm)	Hg (ppm)
TKM Clinics	Galgeun-tang (葛根湯)	0.000	0.007	0.008	0.002
	Kangwhalyupung-tang (羌活愈風湯)	0.002	0.009	0.016	0.002
	Dangguisu-san (當歸鬚散)	0.001	0.010	0.011	0.001
	Dokhwalgisaeng-tang (獨活寄生湯)	0.002	0.018	0.012	0.001
	Banhasasim-tang (半夏瀉心湯)	0.002	0.007	0.006	0.001
	Bangpungtongseong-san (防風通聖散)	0.001	0.005	0.016	0.001
	Bojungikgi-tang (補中益氣湯)	0.001	0.005	0.004	0.001
	Sipjeondaebo-tang (十全大補湯)	0.002	0.003	0.007	0.001
	Ssanghwa-tang (雙和湯)	0.002	0.007	0.006	0.001
	Ojeok-san (五積散)	0.003	0.016	0.008	0.001
TKM Hospitals	Galgeun-tang (葛根湯)	0.001	0.009	0.005	0.001
	Kangwhalyupung-tang (羌活愈風湯)	0.002	0.010	0.015	0.001
	Dangguisu-san (當歸鬚散)	0.002	0.010	0.006	0.001
	Dokhwalgisaeng-tang (獨活寄生湯)	0.002	0.015	0.013	0.001
	Banhasasim-tang (半夏瀉心湯)	0.001	0.007	0.008	0.001
	Bangpungtongseong-san (防風通聖散)	0.001	0.005	0.008	0.001
	Bojungikgi-tang (補中益氣湯)	0.001	0.009	0.001	0.001
	Sipjeondaebo-tang (十全大補湯)	0.001	0.012	0.009	0.001
	Ssanghwa-tang (雙和湯)	0.001	0.012	0.006	0.001
	Ojeok-san (五積散)	0.001	0.012	0.003	0.001
External Herbal Dispensaries	Galgeun-tang (葛根湯)	0.001	0.012	0.001	0.001
	Kangwhalyupung-tang (羌活愈風湯)	0.003	0.013	0.009	0.000
	Dangguisu-san (當歸鬚散)	0.001	0.007	0.008	0.000
	Dokhwalgisaeng-tang (獨活寄生湯)	0.002	0.009	0.004	0.000
	Banhasasim-tang (半夏瀉心湯)	0.001	0.023	0.004	0.000
	Bangpungtongseong-san (防風通聖散)	0.002	0.010	0.000	0.000
	Bojungikgi-tang (補中益氣湯)	0.002	0.008	0.006	0.001
	Sipjeondaebo-tang (十全大補湯)	0.001	0.010	0.007	0.001
	Ssanghwa-tang (雙和湯)	0.003	0.008	ND	0.000
	Ojeok-san (五積散)	0.001	0.012	0.000	0.001
Test criteria		≤0.3 ppm	≤5 ppm	≤3 ppm	≤0.2 ppm
Mean ± SD		0.046 ± 0.0007	0.3 ± 0.004	0.207 ± 0.004	0.026 ± 0.0005

0.000: less than the quantitative limit, ND: less than the detection limit, TKM: Traditional Korean Medicine, External herbal dispensaries: type of pharmacy that provides various types of herbal medicines to other TKM clinics or hospitals. SD: Standard Deviation.

## Data Availability

The data will be made available upon reasonable request.

## Supplementary Materials

The following supporting information can be downloaded at: https://www.mdpi.com/article/10.3390/ijerph19148523/s1, Table S1: 10 types of herbal decoction formulae and compositions from traditional Korean medicine clinics, hospitals, and external herbal dispensaries.
