# Supplementary material for: Monitoring of Heavy Metals and Pesticide Residues of Herbal Decoctions in Traditional Korean Medicine Clinics in Korea
Source: Int J Environ Res Public Health. 2022 Jul 12;19(14):8523. doi: 10.3390/ijerph19148523 (PMC9317733; doi:10.3390/ijerph19148523)
Supplement: Supplementary file 1 [file ijerph-19-08523-s001.zip › ijerph-1742806-supplementary.pdf]

Table S1. 10 types of herbal decoction formulae and compositions from traditional Korean medicine clinics, hospitals, and external herbal dispensaries

	Ssanghwa-tang(雙和湯)	Sipjeondaebo-tang(十全大補湯)	Galgeun-tang(葛根湯)	Bojungikgi-tang(補中益氣湯)	Banhasasim-tang(半夏瀉心湯)	Ojeok-san (五積散)	Dangguisu-san (當歸鬚散)	Bangpungtongseong-san (防風通聖散)	Dokhwalgisaeng-tang (獨活寄生湯)	Kangwhalyupung-tang (羌活愈風湯)
Glycyrrhiza uralensis Fischer (甘草)	○	○	○	○	○	○	○	○	○	○
Angelica gigas Nakai (當歸)	○	○		○		○	○	○	○	○
Paeonia lactiflora Pallas (芍藥)	○	○	○			○	○	○	○	
Zingiber officinale Roscoe (乾薑)	○	○	○	○	○	○				
Cinnamomum cassia Presl (肉桂)	○	○	○				○		○	○
Panax ginseng C. A. Meyer (人參)		○		○	○				○	○
Cnidium officinale Makino (川芎)	○					○		○	○	○
Astragalus membranaceus Bunge (黃耆)	○	○		○						○
Ephedra sinica Stapf (麻黃)			○			○		○		○
Euphorbia pekinensis Ruprecht (大棗)	○		○		○	○				
Zingiber officinale Roscoe (生薑)								○	○	○
Scutellaria baicalensis Georgi (黃芩)					○			○		○
Saposhnikovia divaricata Schischkin (防風)								○	○	○
Rehmanniae radix preparata (熟地黃)	○	○							○	

Poria cocos Wolf (茯苓)	○			○			○	
Atractylodes macrocephala Koidzumi (白朮)	○	○				○		
Bupleurum falcatum Linné (柴胡)		○						○
Fraxinus rhynchophylla Hance (陳皮)		○		○				
Cyperus rotundus Linné (香附子)				○	○			
Platycodon grandiflorum A. De Candolle (桔梗)				○		○		
Pinellia ternata Breitenbach (半夏)			○	○				
Aralia continentalis Kitagawa (獨活)							○	○
Eucommia ulmoides Oliver (杜仲)							○	○
Asiasarum sieboldii Miquel var. seoulense Nakai (細辛)							○	○
Mentha arvensis Linné var. piperascens Malinvaud ex Holmes (薄荷)						○		○
Gypsum Fibrosum(石膏)						○		○
Angelica dahurica Bentham et Hooker f (白芷)				○				○
Atractylodes lancea De Candlle (蒼朮)				○				○
Gentiana macrophylla Pallas (秦艽)							○	○

Pueraria lobata Ohwi (葛根)	○						
Cimicifuga heracleifolia Komarov (升麻)		○					
Coptis japonica Makino (黃連)			○				
Cinnamomum cassia Presl (桂枝)				○			
Poncirus trifoliata Rafinesque (枳實)				○			
Magnolia ovobata Thunberg (厚朴)				○			
Prunus persica Batsch (桃仁)					○		
Caesalpinia sappan Linné (蘇木)					○		
Linderae Radix(烏藥)					○		
Carthamus tinctorius Linné (紅花)					○		
Natrii sulfas(芒硝)						○	
Rheum palmatum Linné (大黃)						○	
Forsythia viridissima Lindley (連翹)						○	
Gardenia jasminoides Ellis (梔子)						○	
Talcum(滑石)						○	
Schizonepeta tenuifolia Briquet (荊芥)						○	
Loranthus parasticus Merr (桑寄生)							○
Achyranthes japonica Nakai (牛膝)							○

Chrysanthemum indicum Linné<br>(甘菊)	○
Ostericum koreanum Maximowicz<br>(羌活)	○
Lycium chinense Miller<br>(枸杞子)	○
Vitex rotundifolia Linné fil<br>(蔓荊子)	○
Paeonia lactiflora Pallas<br>(白芍藥)	○
Rehmannia glutinosa (Gaertner) Liboschitz ex Steudel<br>(生地黄)	○
Citrus aurantium Linné<br>(枳殼)	○
Lycium chinense Miller<br>(地骨皮)	○
Anemarrhena asphodeloides Bunge<br>(知母)	○

○ : The herb is included in the herbal decoction
